# Language Barriers to Online Search Interest for COVID-19: A Global Infodemiological Study

**DOI:** 10.7759/cureus.25574

**Published:** 2022-06-01

**Authors:** Vikram Shee, Christina Louis

**Affiliations:** 1 Physician Case Manager, Teladoc Health, Shanghai, CHN; 2 Medical Director (Southeast Asia), Teladoc Health, Shanghai, CHN

**Keywords:** language, covid-19, coronavirus, infodemiology, infoveillence, big data, google trends, baidu, cross-correlation

## Abstract

Background

Implementation of coronavirus disease 2019 (COVID-19) pandemic control measures requires the engagement and participation of the public in a synchronized manner. Language may be a barrier to captivating public interest in a concerted manner. The relative volume of English and non-English COVID-19-related web searches estimate public interest among English and Non-English “searchers,” respectively. Asynchrony between English and non-English search interest may suggest language-related lapses in public engagement. Addressing these lapses may improve public health communications. In this study, we aimed to describe the distribution and temporal trends in the evolution of English and non-English online search interest for COVID-19 and to identify lags between English and non-English search interest.

Methodology

Search interest data (Baidu Index for China, Google Trends for other countries) was queried for the keywords “coronavirus,” “covid 19,” and their non-English equivalents between January 1, 2019, and September 30, 2020, for each country (n = 230). Daily total, English, and non-English search interest were recorded. Search Interest variables were described at global, regional, and country levels. The cross-correlation function was used to identify lags between English and non-English search interest at global, regional, and country levels.

Results

Globally, 9.69% of total searches relating to COVID-19 utilized non-English keywords. Among included regions, 64.7% (11/17) had significant non-English interest. Central Asia had the highest proportion of non-English interest (81.13% of total interest), followed by Eastern Europe (56.17%), Eastern Asia, Western Asia, and Northern Africa (all over 20%). Among included countries, 33.5% (77/230) had significant non-English interest. Cross-correlation function identified significant lags between English and non-English Interest in six regions (median lag [interquartile range, IQR]: -0.5 [6.00] days) and 24 countries (median lag [IQR]: -1 [4.25] days).

Conclusions

Non-English keywords contribute substantially to searches relating to COVID-19 in certain countries and regions. Numerous locations exhibit significant lags between English and non-English search interest, suggesting language-related discrepancies in the interest for COVID-19. Further research is required to address the root cause of these lags.

## Introduction

The coronavirus disease 2019 (COVID-19) pandemic has resulted in unprecedented morbidity and mortality, as well as unparalleled economic, political, and social losses worldwide. Public health bodies have implemented a plethora of interventions to manage the pandemic [1]. The prompt participation of the public in a concerted manner is required for many of these interventions to be effective.

Google Trends and Baidu Index are search engine analytics tools [2]. They provide a measure of the relative volume of searches (RSVs) for any keyword on a given day. As online searches can be construed as demand for information relating to the search topic, RSVs represent a surrogate measure of public interest in a topic. Several studies have investigated RSVs as a maker of interest in topics pertaining to COVID-19 [3-8].

Thus far, language utilization for COVID-19-related searches has not been explored. An understanding of the distribution of and temporal trends in language utilization may allow optimization of public health communications to the target audience. Furthermore, identifying asynchrony based on search language choice may suggest language-related inefficiencies in communication.

Here, we aim to describe the distribution and temporal trends in the evolution of English and non-English online search interest for COVID-19 at global, regional, and country levels. Furthermore, we hope to identify temporal trends and lags between English and non-English search interest in various countries and regions. Thereby, this research may inform public health officials on regions with inefficient health communications based on differences in language utilization.

## Materials and methods

Preparation of datasets

**Figure 1:**
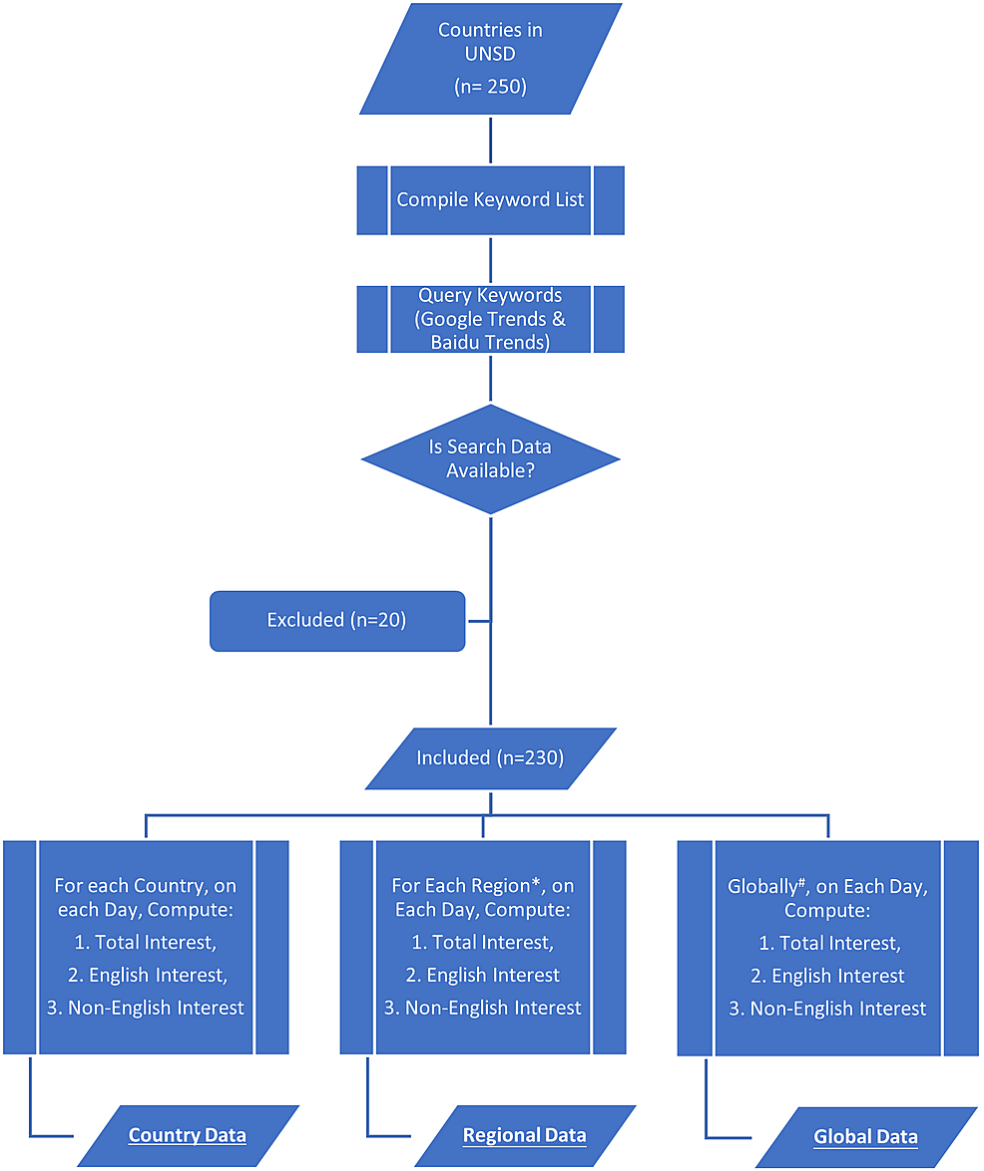
Outline of search data acquisition and processing. *For regional data, the median value of interest variables among countries in each region was calculated for each day. ^#^For global data, the median value of interest variables among all included countries was calculated for each day.

An outline of the acquisition and processing of data is presented in Figure 1.

List of countries and regions

Countries listed by the United Nations Statistics Division (UNSD) were considered for inclusion in this study (n = 250) [9]. Twenty countries were excluded from further analysis due to the unavailability of Google Trends search data. The final analytical cohort included 230 countries. Countries were grouped into regions for further analysis, where regions refer to “sub-regions,” as defined by the UNSD [9].

Compilation of keyword list

English Keywords

Two English keywords relating to COVID-19 were selected. The first, “covid 19,” is the official name for the 2019 coronavirus disease [10]. The second, “coronavirus,” was the most popular keyword referring to the 2019 coronavirus disease worldwide, both before and after the formalization of nomenclature by the World Health Organization (WHO) [10]. Other keywords, as expected, yielded comparatively low interest. The English keywords selected were in keeping with other contemporary studies [3-5].

Non-English Keywords

A reference list of languages spoken in various countries was acquired from data published by the Central Intelligence Agency (United States) [11]. Google Translator (Google, CA, USA) was used to translate English keywords into various non-English language equivalents for each country. English and non-English keywords for each country were compiled into a final keyword list.

Google Trends and Baidu Index data

The measure of search interest provided by Google Trends is the RSV. Google describes how RSVs are calculated as follows:

Google Trends normalizes search data to make comparisons between terms easier. Search results are normalized to the time and location of a query by the following process: Each data point is divided by the total searches of the geography and time range it represents to compare relative popularity. Otherwise, places with the most search volume would always be ranked highest. The resulting numbers are then scaled on a range of 0 to 100 based on a topic’s proportion to all searches on all topics. Different regions that show the same search interest for a term don't always have the same total search volumes [2].

Although Google elaborates on how RSVs are calculated, they do not disclose the absolute search volumes from which RSVs are derived.

Unlike Google Trends, Baidu Index provides time series data of absolute search volumes for keywords queried. For the sake of comparability with Google Trends data, these values were converted into RSVs through the same normalization process used by Google Trends.

Querying search interest data

Search data were queried by specifying the following parameters: (1) Location: each country included in the study; (2) Keywords: country-specific keyword list, as defined above; (3) Time range: study period, i.e., January 1, 2020, to September 30, 2020. Google Trends data were queried using the Pytrends API for Python for all countries except the People’s Republic of China (PRC) [12]. Baidu Index data were queried exclusively for the PRC through the Baidu Index website [13]. Baidu Index data were converted to RSVs according to the normalization process described by Google for comparability with Google Trends data. Countries with no search data were excluded from further analysis (n = 20).

Data processing

Country-Level Data

Daily total interest, English interest, non-English interest, and non-English percentage (i.e., interest variables) were calculated for each country, on each day, as described in Table 1.

Regional and Global Data

**Table 1:** Derivation of search interest variables. ^#^Values for each listed variable were derived from country data and calculated in the same way. Interest variables: total interest, non-English percentage, English interest, non-English interest. day x: represents each day during the study period. Region y: represents each region included in this study.

Interest variables	Method of calculation
Country data	
Unstandardized total interest	Unstandardized total interest_day x_ = English interest_day x_ + Non-English Interest_day x_
Total interest	Unstandardized total interest over the study period normalized on a scale of 0 to 100.
Non-English percentage	Non-English percentage_day x_ = English interest_day x_ ÷ (English interest_day x_ + Non-English interest_day x_)
English interest	English interest_day x_ = Total Interest_day x_ × (1 - Non-English percentage)
Non-English interest	Non-English interest_day x_ = Total interest_day x_ × Non-English percentage
Regional data	
Total interest, non-English percentage, English interest, non-English interest^#^	Interest variable for region y_day x_ = Median interest variable among countries in region y_day x_
Global data	
Total interest, non-English percentage, English interest, non-English interest^#^	Global interest variable_day x_ = Median interest variable among all countries_day x_
Regional English-only data/Regional multi-language data	
Total interest	Total interest for region y_day x_ = Median total interest among countries in region y_day x_
Global English-only data/Global multi-language data	
Total interest	Global total interest_day x_ = Median total interest among all countries_day x_

Regional and global data were derived from the country-level data. For regional data, the median value of interest variables among countries in each region was calculated for each day. For global data, the median values among all included countries were calculated. Table 1 provides further details.

English-Only and Multi-Language Searching Countries

A country or region was considered to have significant non-English interest if the proportion of non-English searches (non-English percentage) on any day during the study period was ≥5%. Countries with significant non-English interest were defined as multi-language searching countries, while the rest were defined as English-only searching countries.

For each region, median values of total interest among English-only and multi-language searching countries within the region were calculated separately. Global total interest among English-only and multi-language searching countries was calculated similarly. Table 1 presents details.

Statistical analysis

Descriptive statistical analyses were performed on Python v3.8.3 using the Jupyter Notebook v6.03.3 [14,15] development environment. Continuous variables appear as medians (interquartile range, IQR). Categorical variables appear as frequencies (%). Maps were created using the QGIS v3.18.1 [16].

Time-series analyses were performed on R Studio v1.2.1335 (Boston, MA, USA). To assess the lags between two time series, cross-correlation function (CCF) analysis and ARIMA modeling were used [17]. The x time series comprised English interest or total interest among English-only searching countries, while the y time series comprised non-English interest or total interest among multi-language searching countries, as specified. First, an ARIMA model was fitted to x using the auto.arima function of the forecast package in R [18]. Subsequently, the y series was filtered with the ARIMA model for x. Finally, CCF analysis was performed between the residuals of x (i.e., pre-whitened x) and filtered y (i.e., transformed y) series. If the highest positive correlation was non-contemporaneous, this suggested the x series lagged or lead y. P-values of <0.05 were considered statistically significant for all analyses.

## Results

Descriptive statistics

**Figure 2:**
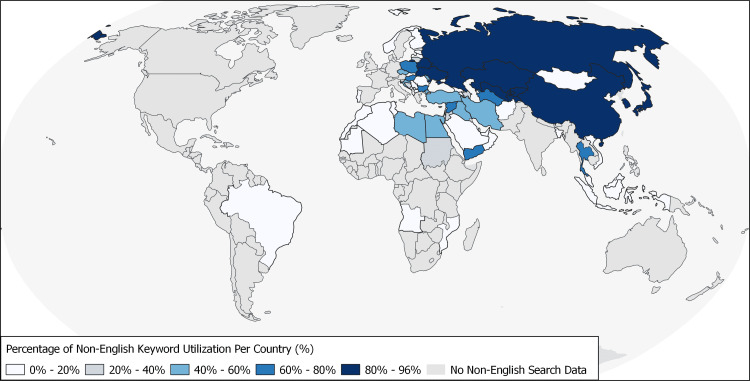
Global distribution of non-English language keyword utilization for COVID-19-related searches. COVID-19: coronavirus disease 2019

Country-level summary statistics are presented in Appendices. In Table 2, summary statistics of global and regional search interest are described. Significant non-English search interest was present in 33.5% (77/230) of countries and 67.7% (11/17) of regions. Summary statistics of global and regional search interest stratified by language (multi-language versus English-only search interest) are shown in Table 3. The geographical distribution of non-English search interest is depicted in Figure 2. Graphical plots of search interest over time (global and regional, Figure 4) and country-level search interest are presented in Appendices (Figures 5-11).

**Table 2:** Descriptive statistics: global and regional search interest. IQR: interquartile range; SD: standard deviation

Location		Average interest, median (IQR)			Non-English %, median (IQR)	Average interest, mean (SD)			Non-English %, mean (SD)
	n	Total	English	Non-English		Total	English	Non-English	
Global		9.47 (8.9)	8.38 (8.36)	0.0 (0.0)	0.0 (0.0)	11.01 (9.97)	9.69 (8.79)	1.23 (1.48)	9.63 (1.86)
Regional									
Australia and New Zealand	2	12.8 (11.03)	12.8 (11.03)	0.0 (0.0)	0.0 (0.0)	17.47 (17.66)	17.47 (17.66)	0.0 (0.0)	0.0 (0.0)
Central Asia	5	10.53 (22.88)	1.55 (2.72)	9.02 (20.52)	86.55 (5.78)	19.25 (17.16)	2.76 (2.74)	16.49 (14.71)	81.13 (18.02)
Eastern Asia	6	12.57 (17.95)	6.25 (7.97)	3.51 (2.83)	45.59 (9.2)	20.04 (15.15)	12.4 (10.15)	7.65 (6.03)	44.75 (6.94)
Eastern Europe	10	11.43 (6.74)	3.68 (2.37)	5.78 (6.34)	67.0 (10.88)	17.0 (16.43)	6.71 (6.11)	10.29 (10.63)	56.17 (15.68)
Latin America and Caribbean	50	10.34 (8.13)	10.34 (8.13)	0.00 (0.00)	0.00 (0.00)	15.94 (15.37)	15.87 (15.3)	0.07 (0.11)	0.27 (0.25)
Melanesia	5	5.25 (6.91)	5.25 (6.91)	0.00 (0.00)	0.00 (0.00)	11.71 (15.84)	11.71 (15.84)	0.00 (0.00)	0.00 (0.00)
Micronesia	7	0.0 (7.17)	0.0 (7.17)	0.00 (0.00)	0.00 (0.00)	8.19 (9.59)	8.19 (9.59)	0.00 (0.00)	0.00 (0.00)
Northern Africa	7	9.64 (9.31)	7.07 (7.82)	1.41 (2.56)	12.31 (18.9)	16.61 (16.32)	11.66 (11.92)	4.96 (4.9)	25.91 (11.3)
Northern America	5	10.37 (12.23)	10.37 (12.23)	0.00 (0.00)	0.00 (0.00)	16.29 (16.89)	16.29 (16.89)	0.00 (0.00)	0.00 (0.00)
Northern Europe	16	10.73 (7.44)	10.11 (6.5)	0.00 (0.00)	0.00 (0.00)	15.73 (15.09)	14.62 (13.83)	1.11 (1.75)	4.94 (3.06)
Polynesia	5	5.37 (12.87)	5.37 (12.87)	0.00 (0.00)	0.00 (0.00)	12.06 (13.57)	12.06 (13.57)	0.00 (0.00)	0.00 (0.00)
Southeastern Asia	11	11.09 (12.11)	10.55 (11.57)	0.00 (0.00)	0.00 (0.00)	18.46 (18.26)	16.53 (16.01)	1.93 (2.61)	9.65 (4.36)
Southern Asia	9	8.72 (17.68)	8.11 (16.85)	0.00 (0.00)	0.00 (0.00)	19.02 (18.8)	17.6 (18.3)	1.42 (1.92)	8.42 (4.33)
Southern Europe	15	12.32 (6.77)	11.18 (6.73)	0.14 (0.15)	0.83 (1.19)	17.17 (16.01)	15.67 (14.6)	1.5 (1.64)	8.28 (3.59)
Sub-Saharan Africa	51	6.01 (10.76)	6.01 (10.76)	0.00 (0.00)	0.00 (0.00)	14.65 (16.63)	14.5 (16.47)	0.15 (0.18)	0.77 (0.65)
Western Asia	18	12.39 (14.12)	10.36 (11.36)	0.87 (1.2)	7.46 (5.57)	19.21 (17.82)	15.67 (15.11)	3.54 (3.04)	20.1 (7.57)
Western Europe	8	10.28 (9.8)	10.28 (9.8)	0.00 (0.00)	0.00 (0.00)	15.94 (16.8)	15.94 (16.8)	0.00 (0.00)	0.00 (0.00)

**Table 3:** Descriptive statistics: global and regional search interest among countries with multi-language and English-only search interest. IQR: interquartile range; SD: standard deviation

Location	n	Total interest, median (IQR)	English interest, median (IQR)	Non-English interest, median (IQR)	Non-English %, median (IQR)	Total interest, mean (SD)	English interest, mean (SD)	Non-English interest, mean (SD)	Non-English %, mean (SD)
Multi-language countries									
Global	77	10.54 (7.85)	7.29 (4.85)	1.15 (2.02)	11.13 (8.76)	12.52 (10.18)	8.66 (6.43)	3.69 (4.41)	28.75 (5.56)
Central Asia	5	10.53 (22.88)	1.55 (2.72)	9.02 (20.52)	86.55 (5.78)	19.25 (17.16)	2.76 (2.74)	16.49 (14.71)	81.13 (18.02)
Eastern Asia	4	10.69 (15.93)	1.52 (1.28)	6.66 (7.75)	85.99 (5.33)	17.58 (13.7)	6.11 (5.61)	11.47 (9.05)	67.12 (10.4)
Eastern Europe	10	11.43 (6.74)	3.68 (2.37)	5.78 (6.34)	67.0 (10.88)	17.0 (16.43)	6.71 (6.11)	10.29 (10.63)	56.17 (15.68)
Latin America and Caribbean	1	13.91 (16.14)	12.54 (12.13)	1.18 (2.6)	9.31 (7.47)	18.21 (17.99)	14.8 (14.57)	3.41 (5.44)	13.53 (12.27)
Northern Africa	7	9.64 (9.31)	7.07 (7.82)	1.41 (2.56)	12.31 (18.9)	16.61 (16.32)	11.66 (11.92)	4.96 (4.9)	25.91 (11.3)
Northern Europe	6	11.49 (9.23)	10.19 (6.58)	0.46(0.91)	4.91 (6.13)	17.87 (16.94)	14.9 (13.57)	2.97 (4.68)	13.16 (8.15)
Southeastern Asia	5	11.06 (11.54)	8.52 (9.19)	1.03 (2.15)	10.09 (10.25)	18.2 (18.58)	13.95 (13.69)	4.24 (5.73)	21.22 (9.6)
Southern Asia	3	8.42 (15.9)	6.95 (14.61)	0.94 (3.33)	8.14 (16.41)	16.88 (17.78)	12.63 (15.55)	4.25 (5.75)	25.22 (13.0)
Southern Europe	10	12.01 (9.84)	10.45 (6.34)	0.36 (0.34)	3.0 (2.19)	16.97 (16.19)	14.73 (14.01)	2.25 (2.46)	12.42 (5.39)
Sub-Saharan Africa	10	5.4 (14.04)	5.22 (14.1)	0.0 (0.11)	0.0 (0.68)	13.77 (15.63)	13.03 (14.78)	0.75 (0.94)	3.95 (3.32)
Western Asia	16	12.01 (13.51)	9.63 (10.1)	1.06 (1.51)	10.54 (7.94)	18.91 (17.52)	14.93 (14.49)	3.98 (3.42)	22.62 (8.51)
English-only countries									
Global	153	8.79 (8.69)	8.79 (8.69)	0.00 (0.00)	0.00 (0.00)	10.18(9.88)	10.18(9.88)	0.00 (0.00)	0.00 (0.00)
Australia and New Zealand	2	12.8 (11.03)	12.8 (11.03)	0.00 (0.00)	0.00 (0.00)	17.47 (17.66)	17.47 (17.66)	0.00 (0.00)	0.00 (0.00)
Eastern Asia	2	17.63 (17.73)	17.63 (17.73)	0.00 (0.00)	0.00 (0.00)	24.97 (20.89)	24.97 (20.89)	0.00 (0.00)	0.00 (0.00)
Latin America and Caribbean	49	10.19 (8.2)	10.19 (8.2)	0.00 (0.00)	0.00 (0.00)	15.89 (15.37)	15.89 (15.37)	0.00 (0.00)	0.00 (0.00)
Melanesia	5	5.25 (6.91)	5.25 (6.91)	0.00 (0.00)	0.00 (0.00)	11.71 (15.84)	11.71 (15.84)	0.00 (0.00)	0.00 (0.00)
Micronesia	7	0.0 (7.17)	0.0 (7.17)	0.00 (0.00)	0.00 (0.00)	8.19 (9.59)	8.19 (9.59)	0.00 (0.00)	0.00 (0.00)
Northern America	5	10.37 (12.23)	10.37 (12.23)	0.00 (0.00)	0.00 (0.00)	16.29 (16.89)	16.29 (16.89)	0.00 (0.00)	0.00 (0.00)
Northern Europe	10	10.44 (8.46)	10.44 (8.46)	0.00 (0.00)	0.00 (0.00)	14.45 (14.24)	14.45 (14.24)	0.00 (0.00)	0.00 (0.00)
Polynesia	5	5.37 (12.87)	5.37 (12.87)	0.00 (0.00)	0.00 (0.00)	12.06 (13.57)	12.06 (13.57)	0.00 (0.00)	0.00 (0.00)
Southeastern Asia	6	11.36 (11.19)	11.36 (11.19)	0.00 (0.00)	0.00 (0.00)	18.67 (18.38)	18.67 (18.38)	0.00 (0.00)	0.00 (0.00)
Southern Asia	6	10.0 (18.88)	10.0 (18.88)	0.00 (0.00)	0.00 (0.00)	20.09 (20.01)	20.08 (20.01)	0.01 (0.02)	0.02 (0.03)
Southern Europe	5	12.35 (9.84)	12.35 (9.84)	0.00 (0.00)	0.00 (0.00)	17.56 (16.67)	17.56 (16.67)	0.00 (0.00)	0.00 (0.00)
Sub-Saharan Africa	41	6.33 (10.76)	6.33 (10.76)	0.00 (0.00)	0.00 (0.00)	14.86 (16.99)	14.86 (16.99)	0.00 (0.00)	0.00 (0.00)
Western Asia	2	14.24 (22.27)	14.24 (22.27)	0.00 (0.00)	0.00 (0.00)	21.61 (21.02)	21.61 (21.02)	0.00 (0.00)	0.00 (0.00)
Western Europe	8	10.28 (9.8)	10.28 (9.8)	0.00 (0.00)	0.00 (0.00)	15.94 (16.8)	15.94 (16.8)	0.00 (0.00)	0.00 (0.00)

The regional and global lag between total interest in English-only and multi-language searching countries

Of the 230 countries included in this study, 77 (33.48%) utilized both English and non-English keywords (multi-language searching countries), while 153 (65.22%) utilized exclusively English keywords (English-only countries). Cross-correlation of total interest between English-only and multi-language searching countries suggested no significant global or regional lags (Table 4).

**Table 4:** Global and regional cross-correlations for total interest between countries with English-only and multi-language searching countries. Dependent variable: median of total interest among English-only searching countries. Independent variable: median of total interest among multi-language searching countries. Cross-correlation was maximum at lag 0 for all regions (bold numbers). ^#^Pearson’s correlation; *p < 0.05. CCF: cross-correlation function

Region	CCF at lag (days)															Correlation at max CCF
	-7	-6	-5	-4	-3	-2	-1	0	1	2	3	4	5	6	7	
Global	0.152*	0.024	-0.031	-0.108	0.105	0.1	0.064	0.758*	0.119	0.052	-0.006	0.103	0.075	0.005	0.19*	0.985
Latin America and Caribbean	-0.099	0.165*	0.189*	0.17*	0.084	0.123*	0.323*	0.536*	-0.011	-0.039	-0.012	-0.038	-0.076	-0.127	-0.04	0.881
Eastern Asia	-0.043	-0.012	-0.08	0.004	0.028	0.004	0.106	0.382*	0.055	0.11	-0.106	-0.027	0.074	0.021	-0.187*	0.744
Northern Europe	-0.153*	0.059	-0.014	-0.086	0.019	0.177*	0.049	0.469*	0.142*	0.078	-0.059	0.103	-0.027	-0.048	-0.066	0.935
Southeastern Asia	0.148*	0.059	-0.069	-0.031	0.053	-0.061	0.141*	0.607*	0.172*	-0.06	0.003	0.024	-0.061	-0.097	-0.102	0.965
Southern Asia	0.19*	0.039	-0.011	-0.008	0.019	-0.041	0.165*	0.354*	0.012	-0.122*	0.036	-0.008	0.008	-0.093	0.177*	0.916
Southern Europe	0.037	0.073	-0.137*	-0.086	0.003	0.158*	0.172*	0.677*	0.264*	0.126*	0.063	-0.041	-0.19*	-0.052	-0.028	0.919
Sub-Saharan Africa	-0.055	0.139*	-0.015	-0.069	0.153*	-0.007	0.205*	0.296*	0.066	0.136*	-0.13*	0.118	0.227*	-0.028	0.218*	0.961
Western Asia	0.067	-0.066	0.065	-0.08	0.077	-0.119	0.119	0.553*	-0.039	0.126*	0.027	0.034	-0.077	0.052	0.052	0.950

Lags between English interest and non-English interest within each country, region, and globally

English and non-English interest were contemporaneous on a global scale. Regionally, language-related asynchrony in search interest was detected in 54.55% (6/11) of regions with significant non-English interest (Table 5). English interest lagged non-English interest in Latin America and Caribbean (one day), Southeastern Asia (one day), and Northern Africa (three days); and led non-English interest in Central Asia (-seven days), Northern Europe (-two days), and Sub-Saharan Africa (-six days). English and non-English interest were contemporaneous in other regions (45.45%, 5/11).

**Table 5:** Global and regional cross-correlations between English and non-English search interest. Dependent variable: median English interest during the study period among multi-language searching countries within region. Independent variable: median non-English interest during the study period among multi-language searching countries within region. ^#^Pearson’s correlation; *p < 0.05. CCF: cross-correlation function

Region	CCF at lags (days)															Lag at max CCF	Correlation at max CCF#
	-7	-6	-5	-4	-3	-2	-1	0	1	2	3	4	5	6	7		
Global	0.024	-0.01	-0.028	-0.06	0.035	0.149*	-0.045	0.438*	0.051	-0.02	0.032	-0.041	0.062	-0.021	0.016	0	0.918
Central Asia	0.217*	0.124*	0.073	-0.155*	0.108	-0.062	0.04	0.197*	0.101	-0.081	0.032	-0.07	0.062	-0.053	0.084	-7	0.801
Eastern Asia	0.049	0.07	-0.018	-0.059	-0.05	0.065	0.188*	0.44*	-0.023	-0.084	0.063	-0.048	-0.031	-0.058	-0.115	0	0.730
Eastern Europe	0.07	-0.02	-0.146*	0.099	0.077	0.23*	0.099	0.291*	0.075	-0.107	0.196*	0.019	-0.081	0.014	-0.083	0	0.841
Latin America and Caribbean	-0.217*	0.07	0.048	-0.106	0.018	0.023	0.055	-0.042	0.133*	0.077	-0.054	0.069	0.026	-0.05	-0.146*	1	0.512
Northern Africa	-0.175*	0.014	0.068	0.025	-0.012	-0.021	-0.064	0.243*	-0.086	0.015	0.325*	-0.187*	0.019	-0.076	0.15*	3	0.811
Northern Europe	0.023	0.11	-0.178*	-0.037	-0.096	0.191*	-0.046	0.101	0.097	-0.042	0.067	-0.011	-0.087	-0.037	-0.021	-2	0.480
Southeastern Asia	-0.107	0.004	-0.005	-0.039	0.02	-0.003	0.112	0.101	0.134*	0.076	-0.019	0.02	-0.071	-0.013	-0.065	1	0.460
Southern Asia	-0.048	-0.065	0.081	0.093	-0.156*	-0.146*	0.099	0.275*	0.129*	-0.123*	0.055	0.002	-0.073	-0.133*	0.263*	0	0.604
Southern Europe	-0.034	-0.072	0.003	-0.098	0.012	0.079	0.126*	0.177*	0.031	0.148*	-0.017	0.063	-0.002	-0.033	-0.098	0	0.779
Sub-Saharan Africa	-0.195*	0.212*	0.046	0.042	0.013	0.11	-0.014	0.158*	0.149*	-0.022	0.023	0.006	0.009	-0.008	-0.011	-6	0.786
Western Asia	-0.071	0.002	-0.008	-0.123*	0.052	0.068	-0.046	0.364*	0.06	-0.136*	0.068	0.088	-0.156*	0.031	0.077	0	0.677

**Figure 3:**
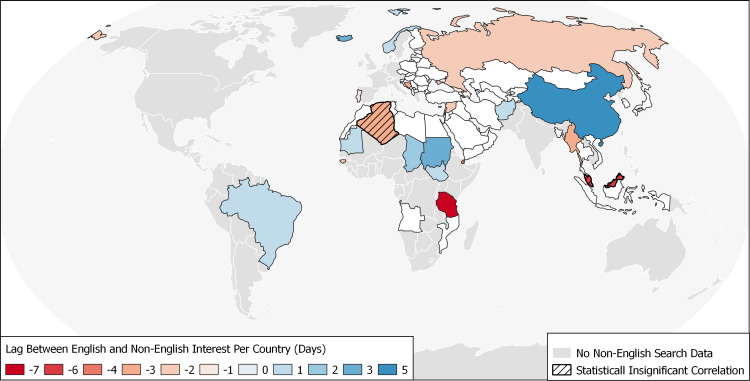
Country-level distribution of lags between English and non-English search interest in COVID-19. Note: A negative lag value may suggest that English interest occurs ahead of (i.e., leads) non-English Interest, while a positive lag value would suggest the converse. COVID-19: coronavirus disease 2019

**Figure 4:**
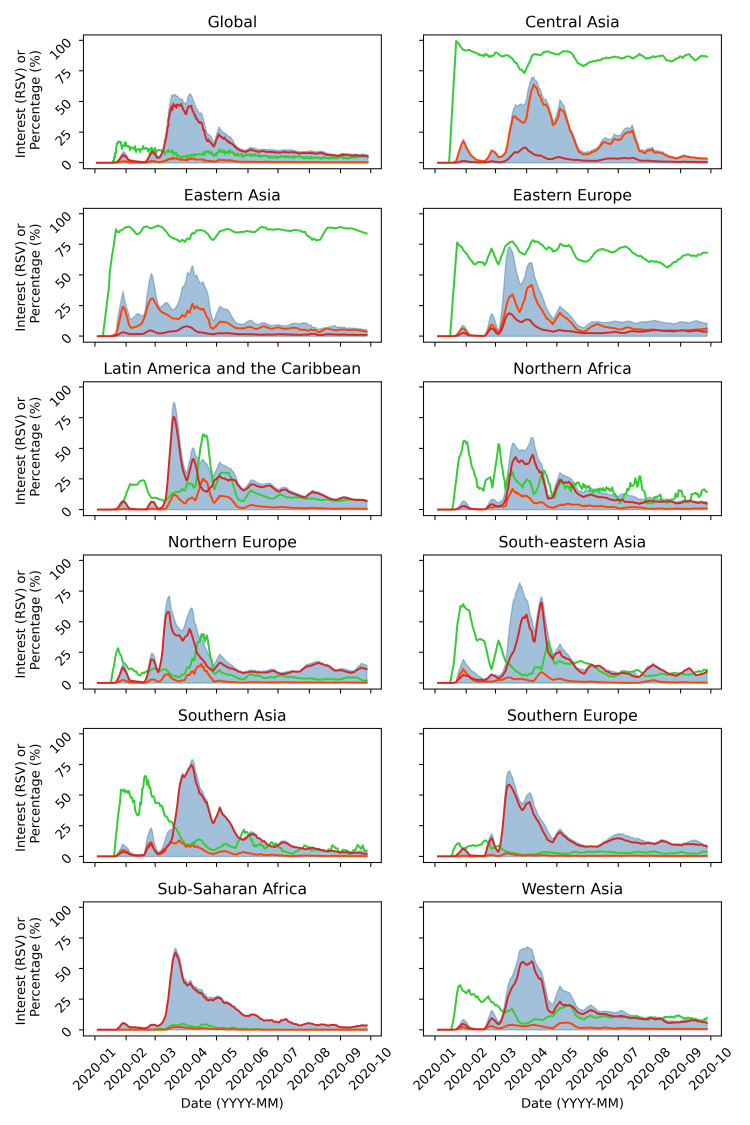
Global and regional trends in search interest for COVID-19 over time. COVID-19: coronavirus disease 2019

Overall, language-related asynchrony in search interest was identified in 31.17% (24/77) of countries with significant non-English interest (Table 6). Specifically, English interest lagged non-English interest in 16.88% (13/77) and led in 14.29% (11/77). Figure 3 illustrates the distribution of lags between English and non-English interest globally.

**Table 6:** Non-contemporaneous cross-correlations between English and non-English interest per country. Dependent variable: median English interest during the study period for each country. Independent variable: median non-English interest during the study period for each country. ^#^Pearson’s correlation; *p < 0.05. CCF: cross-correlation function

Country	-7	-6	-5	-4	-3	-2	-1	0	1	2	3	4	5	6	7	Lag at max CCF	Correlation at max CCF^#^
Afghanistan	-0.054	-0.035	0.067	0.062	-0.137*	-0.157*	0.168*	0.259*	0.265*	-0.055	0.05	-0.078	-0.097	0.016	0.199*	1	0.624
Myanmar	0.012	-0.028	0.094	-0.213*	0.31*	0.121*	0.201*	0.086	0.004	0.026	-0.02	-0.114	-0.048	0.004	-0.061	-3	0.516
Malaysia	-0.11	0.109	-0.076	0.031	-0.038	-0.006	-0.007	0.094	0.044	-0.021	0.025	0.023	-0.019	0.007	0.014	-6	-0.077
China	-0.103	-0.111	-0.116	-0.101	-0.02	-0.056	0.024	0.116	0.187*	0.156*	0.157*	0.219*	0.233*	0.142*	0.123*	5	0.588
State of Palestine	0.051	0.039	-0.034	0.104	-0.121*	0.137*	0.073	0.095	-0.001	0.073	-0.087	-0.013	0.155	-0.047	-0.064	5	0.549
Georgia	-0.008	-0.016	0.102	-0.168*	0.065	-0.016	0.277*	0.233*	-0.063	-0.092	0.099	-0.005	-0.025	-0.098	-0.054	-1	0.902
Andorra	-0.04	-0.053	0.092	-0.018	-0.056	0.012	0.038	-0.007	-0.041	0.014	0.082	-0.069	-0.019	0.037	-0.038	-5	-0.003
Algeria	0.106	0	-0.155*	0.086	0.112	-0.043	0.018	0.022	0.058	0.09	-0.037	-0.048	0.045	0.109	-0.078	-3	0.569
Sudan	-0.012	0.018	-0.021	-0.035	-0.052	0.062	0.142*	0.077	0.071	0.068	0.189*	-0.094	0.157*	-0.037	0.067	3	0.859
Cabo Verde	0.031	-0.013	0.025	-0.025	0.015	0.024	0.1	0.037	0.067	0.083	0.006	-0.163*	-0.109	0.069	0.12*	7	0.448
Chad	0.023	-0.101	0.05	0.016	0.145*	-0.07	0.197*	-0.009	-0.048	0.254*	-0.118	0.118	-0.121*	0.129*	0.093	2	0.616
South Sudan	0.096	-0.156*	-0.056	0.022	0.013	-0.112	-0.094	-0.066	0.224*	0.084	0.009	0.027	0.089	-0.013	0.011	1	0.610
Mauritania	0.035	0.023	0.132*	-0.181*	0.001	-0.019	-0.026	0.04	0.196*	0.06	-0.008	-0.003	0.119	0.149*	-0.035	1	0.651
Guinea-Bissau	-0.115	0.058	0.005	-0.008	-0.151*	0.311*	-0.021	0.065	0.056	0.004	-0.055	0.087	0.024	0.027	0.096	-2	0.613
Syrian Arab Republic	0.096	-0.212*	-0.007	-0.011	-0.117	0.265*	0.041	0.074	0.071	0.028	-0.107	-0.042	-0.037	-0.004	0.041	-2	0.636
Tanzania	0.224*	-0.025	-0.091	-0.092	0.022	0.026	0.048	0.21*	0.006	-0.011	0.046	0.044	0.043	-0.172*	0.024	-7	0.258
Djibouti	-0.09	0.147*	-0.09	0.167*	-0.022	-0.02	-0.037	-0.073	-0.068	-0.001	0.11	-0.003	-0.046	-0.069	0.035	-4	0.279
Iceland	0.031	-0.018	-0.003	-0.046	-0.047	0.045	-0.059	0.168*	-0.034	-0.09	0.189*	-0.048	-0.062	0.054	-0.058	3	0.084
Norway	-0.035	0.058	-0.233*	0.008	0.133*	0.128*	-0.02	-0.197*	0.148*	0.119	-0.023	0.019	-0.226*	0.038	0.043	1	0.325
Portugal	0.037	-0.01	-0.017	-0.11	-0.06	0.221*	0.34*	-0.051	-0.176*	-0.075	0.226*	0.01	0.025	-0.092	0.022	-1	0.835
Bosnia & Herzegovina	0.061	0.002	0.04	-0.178*	0.187*	0.032	-0.056	0.164*	0.019	0	-0.066	0.044	-0.032	-0.002	-0.035	-3	0.647
Malta	0.005	0.188*	-0.085	-0.005	0.12	0.072	0.046	0.033	0.008	-0.005	-0.032	-0.043	-0.085	-0.001	0.003	-6	0.325
Russian Federation	0.012	0.07	0.093	-0.095	-0.092	0.311*	0.091	0.165*	0.082	-0.054	-0.084	0.243*	-0.227*	-0.008	-0.205*	-2	0.910
Brazil	-0.217*	0.07	0.048	-0.106	0.018	0.023	0.055	-0.042	0.133*	0.077	-0.054	0.069	0.026	-0.05	-0.146*	1	0.512

## Discussion

The widespread use of search engines, such as Google and Baidu, to query pandemic-related information using local language keywords has provided new opportunities for health surveillance. Using Google Trends and Baidu Index search analytics data, we identified countries and regions with language-related lags in search interest for the first time. Further research is required to identify the reason for asynchrony in search interest based on the keyword language used. Addressing these issues on a case-by-case basis may improve health communications and result in better implementation of pandemic control policies.

In 2021, Google Search accounted for approximately 92% of the global search engine market share [19]. While Google Search is the predominant search engine in most countries, Baidu Search holds the largest market share in China (75% of the market share). Together, the large user base of Google and Baidu search engines account for their usefulness in epidemiological research.

Previous studies have investigated epidemiological applications of Google Trends and Baidu Index data with regard to the COVID-19 pandemic. Ciaffi et al. demonstrated that symptom searches for keywords such as “fever” and “cough” was associated with intensive care unit (ICU) admissions and deaths related to COVID-19. The premise of this hypothesis was that as patients experienced symptoms, the frequency of Google searches for those symptoms would increase [6]. Using a more generalized approach to keyword selection, Mavragani et al. demonstrated that searches for “coronavirus” are associated with COVID-19 incidence and mortality in the United States [5]. Similar studies also showed significant correlations of keywords with the incidence of COVID-19 cases in other countries as well [3,7]. Husnayain et al. assessed the lag between COVID-19-related searches and the incidence of cases in various provinces of Taiwan [8]. He suggested that search interest can help identify the optimal timing and location for risk communications relating to the pandemic. While applications of search engine data for epidemic surveillance, forecasting, and public health communications relating to COVID-19 have been studied, keyword language utilization has not been explored.

Global distribution of search language

Herein, the worldwide distribution of English and non-English language keyword utilization for COVID-19-related web searches on Google (worldwide) and Baidu (China) search engines is described (Figure 2, Tables 2-4). While interpreting the data, it is important to note that the language chosen for online searches does not always reflect the languages that are predominantly spoken in a specific country. For instance, while Hindi is the most commonly spoken language in India, English keywords are predominantly used for web searches. In addition, English and local language keywords referring to COVID-19 might be identical for several languages (e.g., French).

Our results suggest that non-English search keywords are often used, accounting for 9.69% of total searches relating to COVID-19 globally. Most countries with significant non-English keyword utilization were concentrated around geographically contiguous regions, including Central Asia, Eastern Europe, Eastern Asia, Western Asia, and Northern Africa, among other regions. This data may be utilized to tailor the language of global health communications to local search language preferences.

Temporal trends in search language use

The temporal changes in search interest in various regions and countries are depicted in Figure 4 and Figures 5-11, respectively. Some regions, such as Central Asia, Eastern Asia, and Eastern Europe demonstrated predominantly non-English search utilization throughout the course of this study.

**Figure 5:**
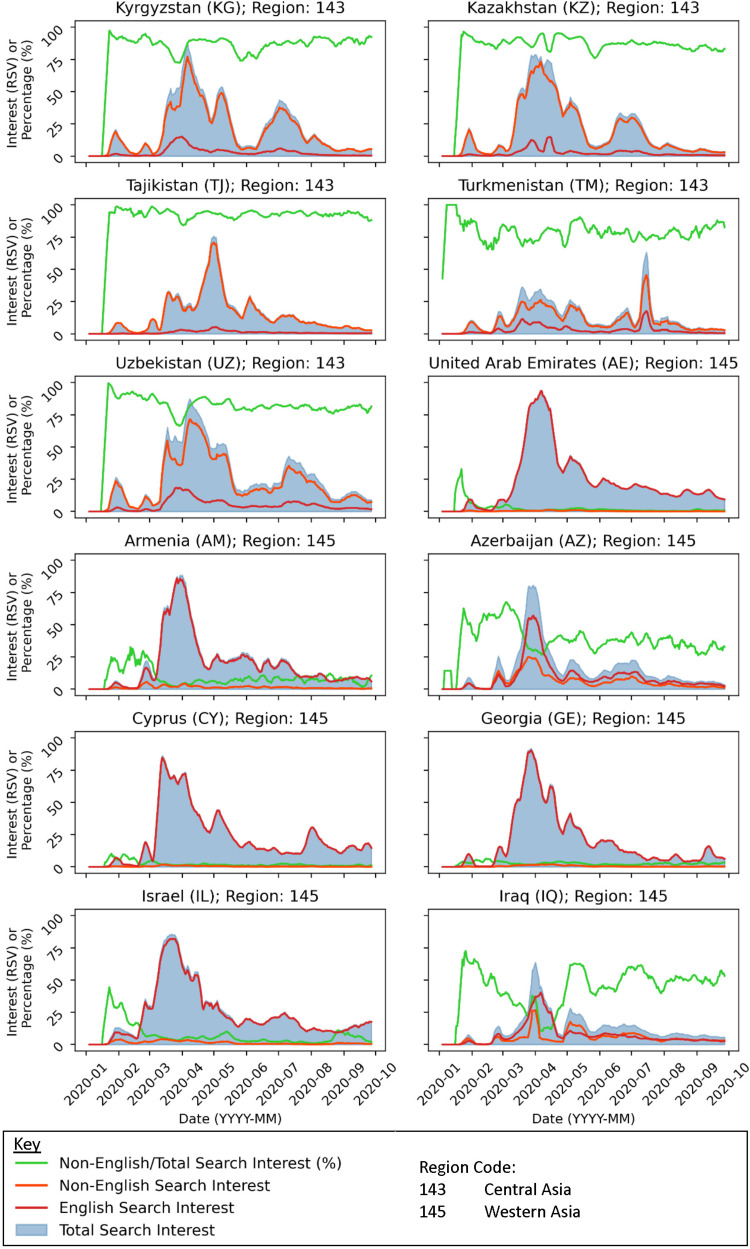
Country-level trends in search interest for COVID-19 over time: part 1. The figure shows temporal changes in total, English, and non-English search interest for COVID-19, and the non-English percentage between January 1, 2020, and September 30, 2020. COVID-19: coronavirus disease 2019

**Figure 6:**
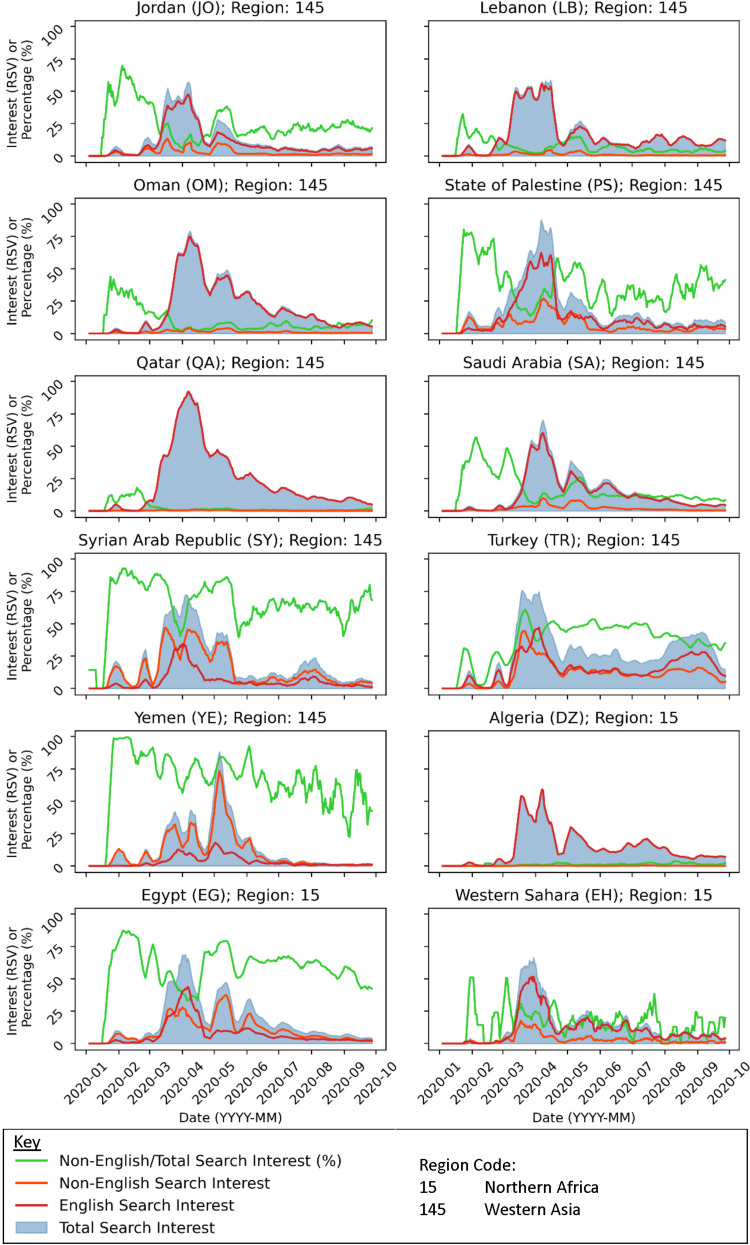
Country-level trends in search interest for COVID-19 over time: part 2. The figure shows temporal changes in total, English, and non-English search interest for COVID-19, and the non-English percentage between January 1, 2020, and September 30, 2020. COVID-19: coronavirus disease 2019

**Figure 7:**
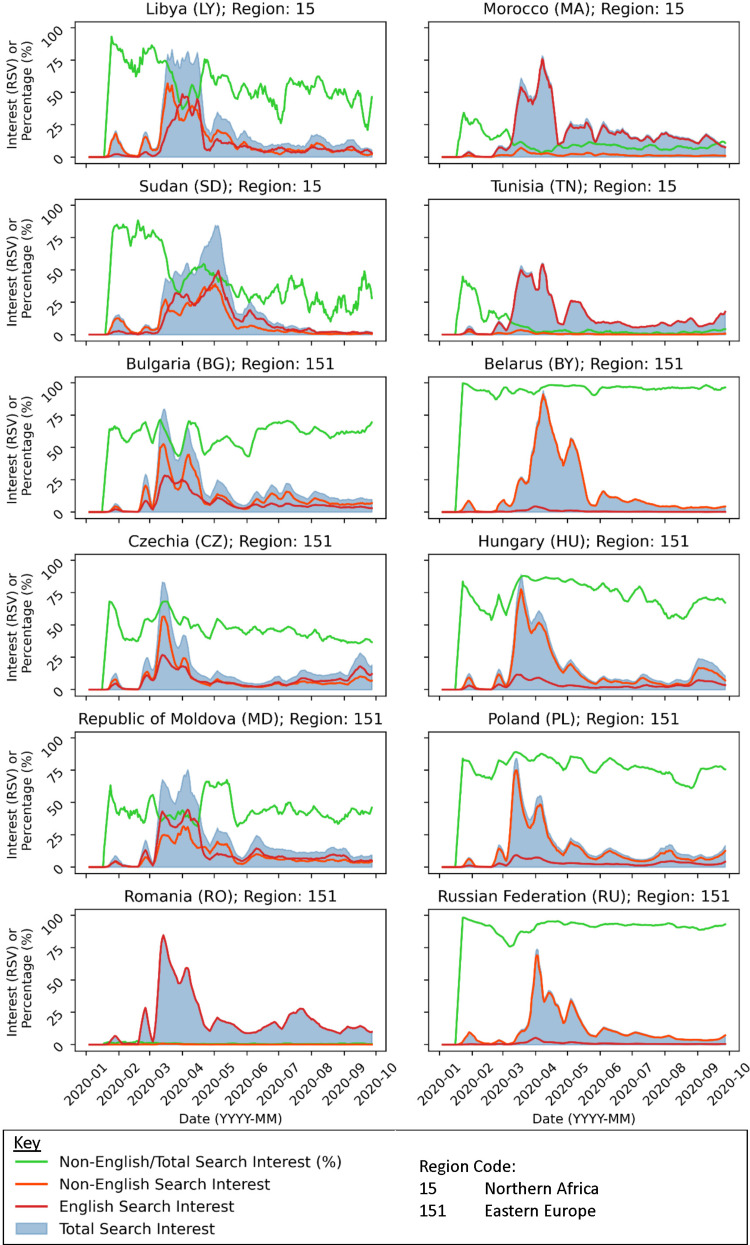
Country-level trends in search interest for COVID-19 over time: part 3. The figure shows temporal changes in total, English, and non-English search interest for COVID-19, and the non-English percentage between January 1, 2020, and September 30, 2020. COVID-19: coronavirus disease 2019

**Figure 8:**
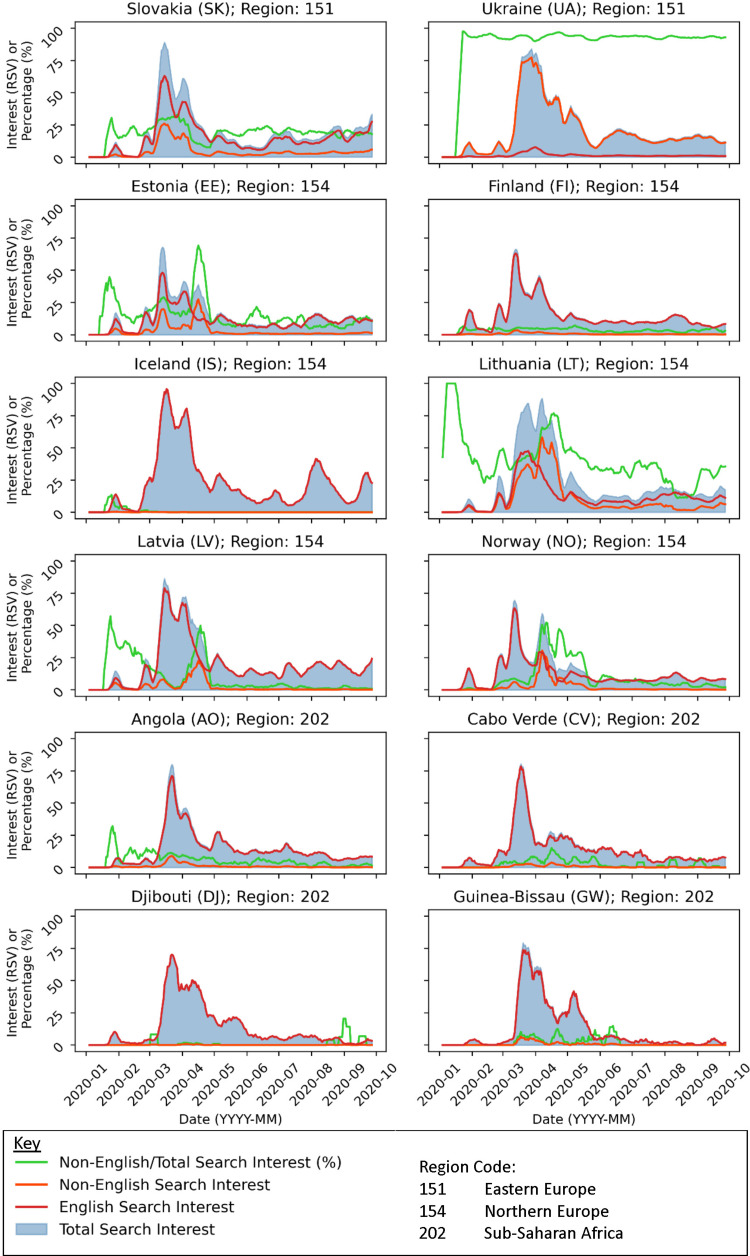
Country-level trends in search interest for COVID-19 over time: part 4. The figure shows temporal changes in total, English, and non-English search interest for COVID-19, and the non-English percentage between January 1, 2020, and September 30, 2020. COVID-19: coronavirus disease 2019

**Figure 9:**
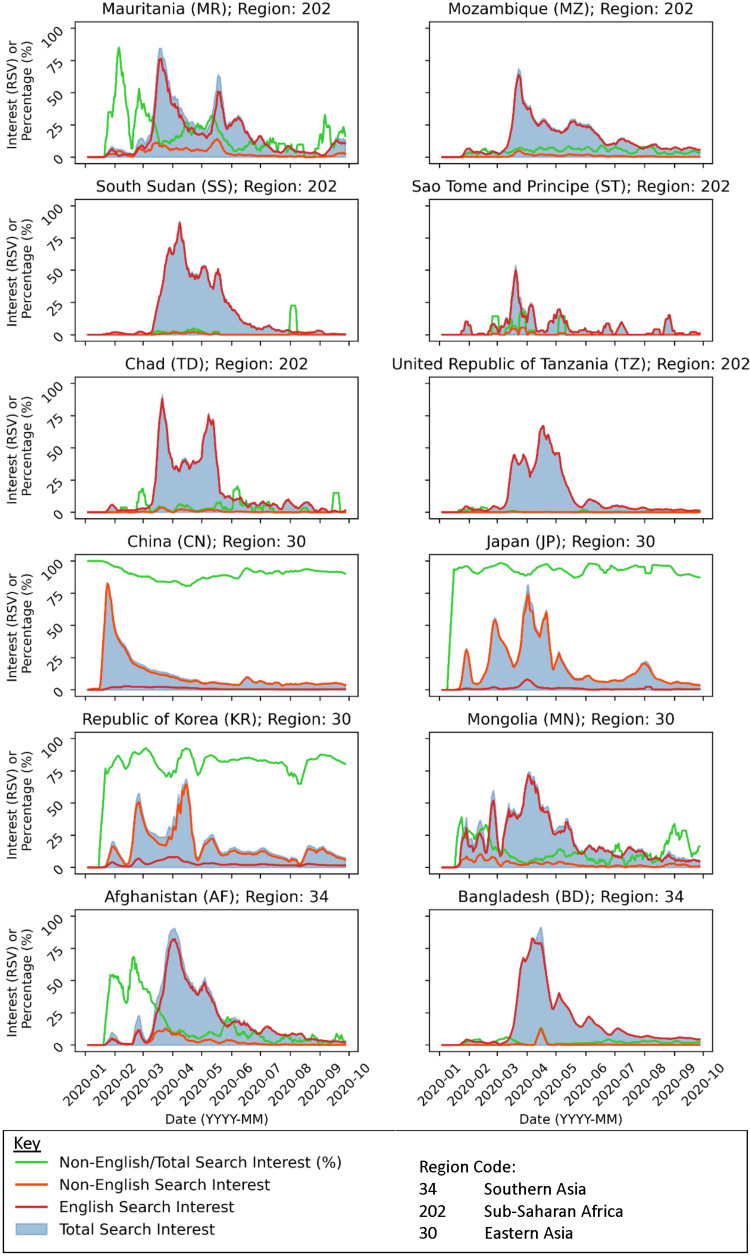
Country-level trends in search interest for COVID-19 over time: part 5. The figure shows temporal changes in total, English, and non-English search interest for COVID-19, and the non-English percentage between January 1, 2020, and September 30, 2020. COVID-19: coronavirus disease 2019

**Figure 10:**
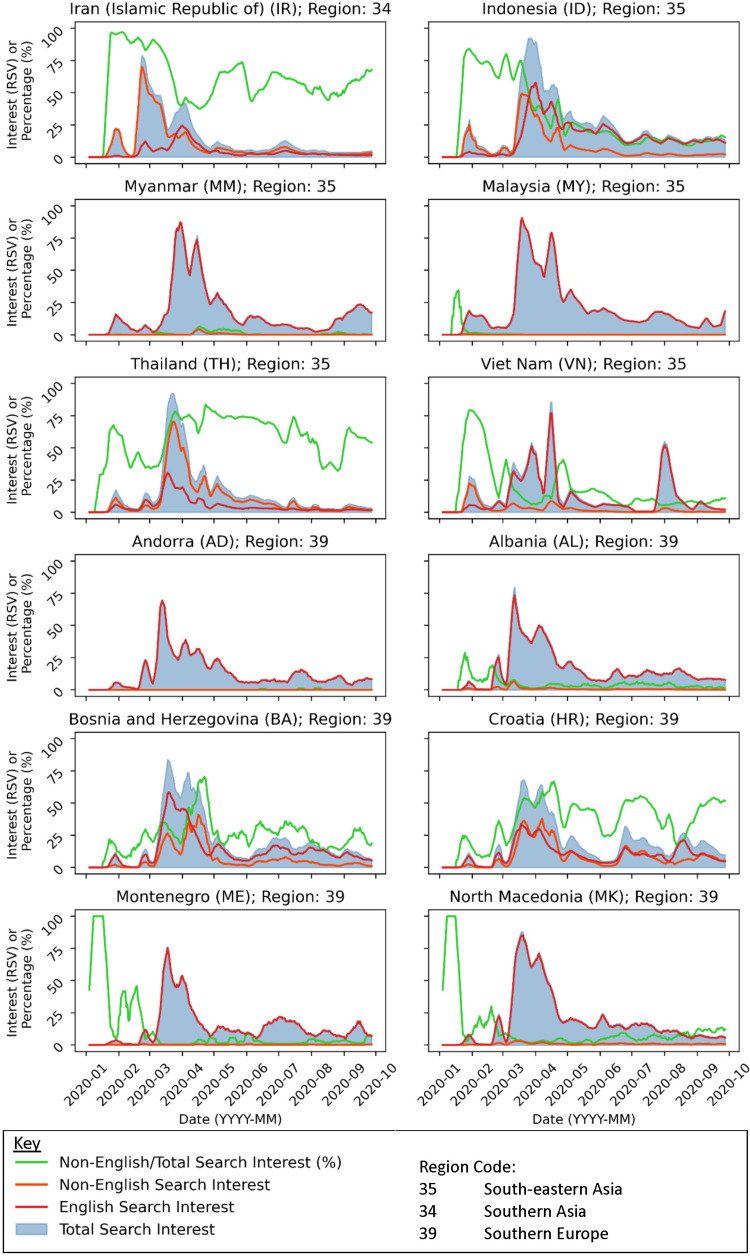
Country-level trends in search interest for COVID-19 over time: part 6. The figure shows temporal changes in total, English, and non-English search interest for COVID-19, and the non-English percentage between January 1, 2020, and September 30, 2020. COVID-19: coronavirus disease 2019

**Figure 11:**
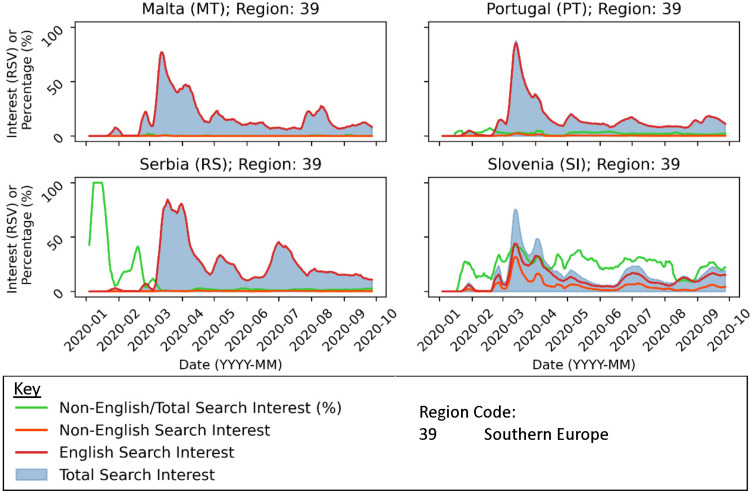
Country-level trends in search interest for COVID-19 over time: part 7. The figure shows temporal changes in total, English, and non-English search interest for COVID-19, and the non-English percentage between January 1, 2020, and September 30, 2020. COVID-19: coronavirus disease 2019

Interestingly, Northern Africa, Southeastern Asia, Southern Asia, and Western Asia showed a high percentage of non-English language searches early in the pandemic, peaking between January and February 2020, followed by a precipitous decline. Referring to individual country-level plots for search interest in each respective region (Figures 5-11), it is apparent that many but not all countries within these regions displayed this pattern. Therefore, regional generalization should be interpreted carefully.

Speculatively, early in the pandemic, there may have been a sudden increase in the demand for information without knowledge of the most appropriate keywords to use. Once the public was educated on appropriate English keywords by the media, government agencies, and other sources, their search habits might have changed. The standardization of nomenclature for the 2019 coronavirus disease by the WHO on February 11, 2020, might have also contributed to the change in search language preferences. These trends bear important implications for future global health emergencies. The early definition of standard terminology in multiple languages is essential to direct the sudden increase in the demand for information to appropriate resources.

Interpretation of cross-correlation coefficients

The CCF analyzes the similarity between a pair of time series when one time series is displaced against the other. One drawback of CCF is that real-world data may suffer from autocorrelation resulting in spurious cross-correlations. This is tackled by removing the autocorrelated component from the input series using a process called pre-whitening. Details are described in the statistics section and are elaborated on in authoritative textbooks [17]. Considering the example of daily English and non-English search interest data, these time series should be contemporaneous, that is, the highest correlation should be at lag 0. A negative lag value may suggest that English interest occurs ahead of (i.e., leads) non-English Interest, while a positive lag value would suggest the converse.

Lags Between Total Interest of English-Only and Multi-Language Searching Countries

Reassuringly, no lags in total interest were found between English-only and multi-language searching countries within any region or globally (Table 4).

Regional, Global, and Country-Level Lags Between English and Non-English Search Interest

It is concerning that the interest of English and non-English searching subpopulations within several countries and regions were not contemporaneous (as depicted in Figure 3 and Tables 5, 6). While multiple factors may contribute to these findings, they are likely to differ on a case-by-case basis.

Delayed communications between languages: Speculatively, delayed communications in a specific language might result in a lagged rise in search interest for the same language. For instance, the news reported in one language might lag reporting in another.

Varying impact of communications between languages: Also, the number of English versus local language media outlets (online, televised, or physical), their viewership, and their impact may vary, thus resulting in the asynchronous public interest.

Intrinsic subpopulation characteristics: The baseline characteristics of individuals searching with English and non-English keywords may vary. Differences in education, socio-economic strata, access to the internet, or other factors might result in a delayed reaction to public health communications, even if communications are delivered in appropriate languages and in a timely manner.

Further research is required to identify the reason for language-related lags and develop interventions to remedy these issues. Neglecting lapses in communication may hamper pandemic control measures and put vulnerable subpopulations at a greater risk.

Limitations

This study has certain limitations that merit consideration. First, although Google (92%) and Baidu (1.3%) represent most online searches worldwide, the exclusion of data from other search engines may lead to bias [19]. Second, approximately 36% of the global population does not have access to the internet in 2021. These individuals cannot be represented through search data. Third, country-level data may represent averages for large and heterogeneous populations. Further research at the sub-country level is indicated. Despite the limitations of search engine data, infodemiological metrics have received wide attention for assisting with public health policy and monitoring epidemics.

## Conclusions

Non-English keywords contribute substantially to searches relating to COVID-19 in certain countries and regions. Numerous locations exhibit significant lags between English and non-English search interest, suggesting language-related discrepancies in the interest for COVID-19. Further research is required to address the root cause of these lags.

## Appendices

**Table 7:** Descriptive statistics: country-level search interest. Note: Interest variables expressed as median (IQR)

Country code	Country name	Region name	Total interest	English interest	Non-English interest	Non-English (%)	Max non-English (%)
Countries with significant foreign language keyword utilization (max non-English (%) ≥ 5%)							
KZ	Kazakhstan	Central Asia	8.84 (26.94)	1.32 (2.3)	7.72 (24.28)	87.30 (7.41)	100.00
KG	Kyrgyzstan	Central Asia	11.11 (28.79)	1.54 (3.59)	10.09 (25.32)	88.66 (8.91)	100.00
TJ	Tajikistan	Central Asia	9.38 (16.09)	0.6 (1.19)	8.61 (14.74)	93.21 (6.16)	100.00
TM	Turkmenistan	Central Asia	8.93 (14.72)	1.74 (2.66)	7.24 (11.84)	79.25 (13.70)	100.00
UZ	Uzbekistan	Central Asia	18.88 (30.59)	3.5 (5.15)	15.82 (24.46)	82.16 (8.08)	100.00
CN	China	Eastern Asia	6.05 (7.89)	0.58 (1.32)	5.4 (6.51)	90.60 (5.86)	100.00
JP	Japan	Eastern Asia	9.5 (18.39)	0.63 (1.0)	8.77 (17.37)	94.06 (6.15)	100.00
MN	Mongolia	Eastern Asia	14.45 (24.86)	12.59 (21.58)	1.82 (3.42)	9.21 (18.31)	87.55
KR	Republic of Korea	Eastern Asia	13.02 (14.6)	2.1 (1.56)	10.87 (10.64)	82.71 (8.91)	100.00
BY	Belarus	Eastern Europe	6.4 (13.22)	0.28 (0.54)	6.11 (12.86)	95.96 (3.61)	100.00
BG	Bulgaria	Eastern Europe	11.81 (12.02)	4.58 (4.6)	7.04 (9.08)	60.03 (14.52)	75.75
CZ	Czechia	Eastern Europe	10.73 (10.68)	5.87 (5.76)	4.68 (5.09)	44.46 (12.47)	70.63
HU	Hungary	Eastern Europe	9.06 (14.64)	2.53 (2.9)	6.58 (11.43)	71.92 (17.54)	100.00
PL	Poland	Eastern Europe	10.17 (10.71)	2.44 (2.08)	7.39 (9.0)	77.71 (11.49)	90.91
MD	Republic of Moldova	Eastern Europe	12.62 (15.44)	7.26 (5.75)	5.24 (8.37)	41.39 (14.55)	100.00
RO	Romania	Eastern Europe	13.48 (13.86)	13.38 (13.74)	0.07 (0.11)	0.01 (0.01)	7.60
RU	Russian Federation	Eastern Europe	7.11 (8.26)	0.52 (0.56)	6.54 (7.86)	92.36 (3.85)	99.15
SK	Slovakia	Eastern Europe	15.21 (14.18)	12.3 (11.62)	2.83 (2.7)	19.01 (7.41)	49.71
UA	Ukraine	Eastern Europe	14.97 (10.42)	0.98 (0.63)	13.97 (9.91)	93.29 (2.41)	99.03
BR	Brazil	Latin America and Caribbean	13.91 (16.14)	12.54 (12.13)	1.18 (2.6)	9.31 (7.47)	65.97
DZ	Algeria	Northern Africa	9.66 (12.38)	9.51 (12.05)	0.13 (0.25)	0.95 (1.68)	13.55
EG	Egypt	Northern Africa	10.13 (15.91)	3.69 (6.18)	6.51 (9.45)	59.35 (20.35)	92.01
LY	Libya	Northern Africa	11.67 (16.89)	5.32 (6.6)	6.15 (12.24)	55.72 (27.34)	100.00
MA	Morocco	Northern Africa	14.95 (13.9)	13.77 (13.44)	1.22 (1.14)	8.33 (4.72)	45.90
SD	Sudan	Northern Africa	7.17 (22.98)	3.04 (13.59)	3.25 (10.89)	38.88 (30.82)	100.00
TN	Tunisia	Northern Africa	8.77 (9.33)	8.54 (9.37)	0.2 (0.46)	2.11 (4.16)	69.39
EH	Western Sahara	Northern Africa	9.12 (13.54)	7.13 (10.42)	0.0 (4.47)	0.00 (27.83)	100.00
EE	Estonia	Northern Europe	10.57 (10.54)	9.51 (8.57)	0.98 (1.65)	11.06 (13.42)	100.00
FI	Finland	Northern Europe	10.25 (7.17)	10.01 (7.17)	0.31 (0.5)	3.36 (3.08)	15.03
IS	Iceland	Northern Europe	15.14 (21.53)	15.14 (21.53)	0.0 (0.0)	0.0 (0.0)	41.27
LV	Latvia	Northern Europe	15.89 (12.79)	15.05 (12.71)	0.36 (0.79)	2.74 (10.43)	100.00
LT	Lithuania	Northern Europe	13.84 (11.36)	9.46 (8.03)	4.25 (5.56)	37.37 (17.76)	100.00
NO	Norway	Northern Europe	8.93 (9.88)	8.14 (6.55)	0.44 (1.23)	4.66 (6.87)	79.37
ID	Indonesia	Southeastern Asia	15.49 (15.99)	13.2 (16.31)	2.89 (5.18)	17.88 (39.52)	87.34
MY	Malaysia	Southeastern Asia	14.4 (15.28)	14.38 (14.96)	0.01 (0.02)	0.03 (0.16)	100.00
MM	Myanmar	Southeastern Asia	10.31 (15.47)	10.17 (15.19)	0.0 (0.13)	0.00 (0.69)	8.73
TH	Thailand	Southeastern Asia	7.77 (12.44)	2.61 (3.43)	4.29 (8.6)	61.18 (29.81)	86.95
VN	Viet Nam	Southeastern Asia	6.51 (14.05)	5.36 (9.01)	1.11 (2.53)	10.15 (10.71)	81.44
AF	Afghanistan	Southern Asia	9.53 (21.67)	8.39 (19.03)	0.94 (3.52)	7.76 (19.14)	100.00
BD	Bangladesh	Southern Asia	6.85 (14.14)	6.63 (13.94)	0.14 (0.23)	1.69 (2.03)	16.16
IR	Iran (Islamic Republic of)	Southern Asia	6.02 (9.6)	2.43 (2.99)	3.75 (5.52)	59.87 (24.28)	98.56
AL	Albania	Southern Europe	11.07 (10.62)	10.62 (10.06)	0.37 (0.6)	2.97 (3.61)	50.98
AD	Andorra	Southern Europe	8.05 (12.39)	8.05 (12.39)	0.0 (0.0)	0.0 (0.0)	9.27
BA	Bosnia and Herzegovina	Southern Europe	13.11 (14.68)	9.88 (9.41)	2.92 (4.87)	22.79 (17.12)	76.56
HR	Croatia	Southern Europe	15.02 (17.72)	8.69 (8.44)	5.49 (9.41)	41.39 (27.92)	73.72
MT	Malta	Southern Europe	10.52 (12.64)	10.52 (12.45)	0.0 (0.0)	0.0 (0.0)	9.57
ME	Montenegro	Southern Europe	10.06 (13.1)	9.7 (12.81)	0.18 (0.0)	1.96 (4.43)	100.00
MK	North Macedonia	Southern Europe	13.05 (13.8)	12.4 (13.76)	0.66 (0.93)	5.51 (7.26)	100.00
PT	Portugal	Southern Europe	11.26 (7.84)	10.86 (7.71)	0.25 (0.28)	2.24 (1.70)	21.26
RS	Serbia	Southern Europe	16.14 (19.57)	15.87 (19.35)	0.27 (0.22)	1.73 (2.33)	100.00
SI	Slovenia	Southern Europe	12.41 (14.4)	9.45 (10.48)	3.13 (4.74)	24.89 (13.03)	64.03
AO	Angola	Sub-Saharan Africa	10.57 (10.74)	10.15 (9.84)	0.41 (0.89)	3.94 (7.89)	57.63
CV	Cabo Verde	Sub-Saharan Africa	8.98 (12.94)	8.73 (12.04)	0.0 (0.76)	0.00 (3.81)	27.39
TD	Chad	Sub-Saharan Africa	5.73 (15.58)	5.65 (15.68)	0.0 (0.0)	0.00 (0.00)	100.00
DJ	Djibouti	Sub-Saharan Africa	6.03 (13.39)	6.03 (13.56)	0.0 (0.0)	0.00 (0.00)	100.00
GW	Guinea-Bissau	Sub-Saharan Africa	3.19 (11.49)	3.19 (11.37)	0.0 (0.0)	0.00 (0.00)	41.71
MR	Mauritania	Sub-Saharan Africa	11.15 (22.08)	9.33 (18.06)	1.81 (5.34)	10.84 (25.17)	100.00
MZ	Mozambique	Sub-Saharan Africa	9.52 (16.91)	9.09 (15.87)	0.49 (1.13)	4.54 (5.30)	16.07
ST	Sao Tome and Principe	Sub-Saharan Africa	0.0 (6.98)	0.0 (6.37)	0.0 (0.0)	0.00 (0.00)	100.00
SS	South Sudan	Sub-Saharan Africa	3.51 (26.15)	3.47 (26.02)	0.0 (0.0)	0.00 (0.00)	100.00
TZ	United Republic of Tanzania	Sub-Saharan Africa	3.34 (8.63)	3.31 (8.66)	0.0 (0.0)	0.00 (0.00)	24.28
AM	Armenia	Western Asia	13.01 (16.93)	12.01 (15.85)	1.01 (1.16)	6.13 (7.35)	71.15
AZ	Azerbaijan	Western Asia	9.58 (13.75)	5.89 (7.69)	4.03 (6.09)	38.19 (15.69)	100.00
CY	Cyprus	Western Asia	15.4 (18.83)	15.17 (18.33)	0.21 (0.36)	1.32 (1.42)	24.95
GE	Georgia	Western Asia	10.32 (19.89)	10.11 (19.25)	0.22 (0.66)	2.28 (1.60)	11.74
IQ	Iraq	Western Asia	8.58 (8.7)	4.59 (4.74)	3.73 (3.67)	46.02 (23.89)	100.00
IL	Israel	Western Asia	17.2 (16.18)	16.12 (15.63)	0.77 (1.76)	4.00 (4.97)	64.29
JO	Jordan	Western Asia	7.22 (7.95)	5.63 (6.77)	1.52 (1.55)	20.17 (18.00)	100.00
LB	Lebanon	Western Asia	11.36 (9.94)	10.59 (9.48)	0.56 (0.93)	4.77 (6.69)	51.92
OM	Oman	Western Asia	13.16 (23.16)	12.41 (22.9)	0.69 (1.2)	5.30 (7.08)	100.00
QA	Qatar	Western Asia	12.56 (26.64)	12.53 (26.5)	0.11 (0.32)	0.79 (1.29)	28.47
SA	Saudi Arabia	Western Asia	9.24 (15.36)	8.29 (12.96)	1.25 (2.22)	11.83 (10.87)	62.21
PS	State of Palestine	Western Asia	10.96 (16.59)	7.08 (9.14)	3.76 (6.28)	31.82 (29.04)	100.00
SY	Syrian Arab Republic	Western Asia	10.62 (20.71)	3.49 (5.25)	7.67 (15.96)	69.30 (26.44)	100.00
TR	Turkey	Western Asia	24.55 (21.66)	13.26 (12.17)	11.05 (9.98)	40.53 (18.35)	63.42
AE	United Arab Emirates	Western Asia	17.08 (15.8)	16.93 (15.41)	0.17 (0.37)	1.02 (1.52)	100.00
YE	Yemen	Western Asia	5.54 (17.02)	1.44 (4.29)	3.43 (13.31)	71.68 (36.37)	100.00
Countries with insignificant foreign language keyword utilization (max non-English (%) < 5%)							
AU	Australia	Australia and New Zealand	13.89 (14.65)	13.89 (14.65)	0.00 (0.00)	0.00 (0.00)	0.00
NZ	New Zealand	Australia and New Zealand	9.53 (11.13)	9.53 (11.13)	0.00 (0.00)	0.00 (0.00)	0.00
HK	China, Hong Kong Special Administrative Region	Eastern Asia	19.78 (21.9)	19.78 (21.9)	0.00 (0.00)	0.00 (0.00)	0.00
MO	China, Macao Special Administrative Region	Eastern Asia	14.53 (22.58)	14.53 (22.58)	0.00 (0.00)	0.00 (0.00)	0.00
AI	Anguilla	Latin America and Caribbean	0.0 (0.0)	0.0 (0.0)	0.00 (0.00)	0.00 (0.00)	0.00
AG	Antigua and Barbuda	Latin America and Caribbean	5.93 (12.27)	5.93 (12.27)	0.00 (0.00)	0.00 (0.00)	0.00
AR	Argentina	Latin America and Caribbean	18.79 (10.92)	18.79 (10.92)	0.00 (0.00)	0.00 (0.00)	0.00
AW	Aruba	Latin America and Caribbean	13.56 (20.7)	13.56 (20.7)	0.00 (0.00)	0.00 (0.00)	0.00
BS	Bahamas	Latin America and Caribbean	11.3 (13.72)	11.3 (13.72)	0.00 (0.00)	0.00 (0.00)	0.00
BB	Barbados	Latin America and Caribbean	10.15 (14.07)	10.15 (14.07)	0.00 (0.00)	0.00 (0.00)	0.00
BZ	Belize	Latin America and Caribbean	9.48 (12.97)	9.48 (12.97)	0.00 (0.00)	0.00 (0.00)	0.00
BO	Bolivia (Plurinational State of)	Latin America and Caribbean	23.97 (29.21)	23.97 (29.21)	0.00 (0.00)	0.00 (0.00)	0.00
BQ	Bonaire, Sint Eustatius and Saba	Latin America and Caribbean	6.58 (16.0)	6.58 (16.0)	0.00 (0.00)	0.00 (0.00)	0.00
VG	British Virgin Islands	Latin America and Caribbean	6.12 (16.24)	6.12 (16.24)	0.00 (0.00)	0.00 (0.00)	0.00
KY	Cayman Islands	Latin America and Caribbean	10.2 (17.32)	10.2 (17.32)	0.00 (0.00)	0.00 (0.00)	0.00
CL	Chile	Latin America and Caribbean	11.16 (15.27)	11.16 (15.27)	0.00 (0.00)	0.00 (0.00)	0.00
CO	Colombia	Latin America and Caribbean	15.21 (13.2)	15.21 (13.2)	0.00 (0.00)	0.00 (0.00)	0.00
CR	Costa Rica	Latin America and Caribbean	14.64 (13.36)	14.64 (13.36)	0.00 (0.00)	0.00 (0.00)	0.00
CU	Cuba	Latin America and Caribbean	11.31 (11.2)	11.31 (11.2)	0.00 (0.00)	0.00 (0.00)	0.00
CW	Curacao	Latin America and Caribbean	9.1 (13.24)	9.1 (13.24)	0.00 (0.00)	0.00 (0.00)	0.00
DM	Dominica	Latin America and Caribbean	5.73 (13.76)	5.73 (13.76)	0.00 (0.00)	0.00 (0.00)	0.00
DO	Dominican Republic	Latin America and Caribbean	11.31 (13.46)	11.31 (13.46)	0.00 (0.00)	0.00 (0.00)	0.00
EC	Ecuador	Latin America and Caribbean	13.97 (18.03)	13.97 (18.03)	0.00 (0.00)	0.00 (0.00)	0.00
SV	El Salvador	Latin America and Caribbean	9.56 (13.08)	9.56 (13.08)	0.00 (0.00)	0.00 (0.00)	0.00
FK	Falkland Islands (Malvinas)	Latin America and Caribbean	0.00 (0.00)	0.00 (0.00)	0.00 (0.00)	0.0 (0.0)	0.00
GF	French Guiana	Latin America and Caribbean	8.25 (12.95)	8.25 (12.95)	0.00 (0.00)	0.00 (0.00)	0.00
GD	Grenada	Latin America and Caribbean	8.68 (15.75)	8.68 (15.75)	0.00 (0.00)	0.00 (0.00)	0.00
GP	Guadeloupe	Latin America and Caribbean	7.11 (10.56)	7.11 (10.56)	0.00 (0.00)	0.00 (0.00)	0.00
GT	Guatemala	Latin America and Caribbean	13.56 (15.68)	13.56 (15.68)	0.00 (0.00)	0.00 (0.00))	0.00
GY	Guyana	Latin America and Caribbean	9.65 (14.5)	9.65 (14.5)	0.00 (0.00)	0.00 (0.00)	0.00
HT	Haiti	Latin America and Caribbean	9.41 (35.36)	9.41 (35.36)	0.00 (0.00)	0.00 (0.00))	0.00
HN	Honduras	Latin America and Caribbean	15.28 (19.12)	15.28 (19.12)	0.00 (0.00)	0.00 (0.00)	0.00
JM	Jamaica	Latin America and Caribbean	10.96 (21.4)	10.96 (21.4)	0.00 (0.00)	0.00 (0.00)	0.00
MQ	Martinique	Latin America and Caribbean	7.92 (10.24)	7.92 (10.24)	0.00 (0.00)	0.00 (0.00)	0.00
MX	Mexico	Latin America and Caribbean	17.36 (16.71)	17.36 (16.71)	0.00 (0.00)	0.00 (0.00)	0.00
MS	Montserrat	Latin America and Caribbean	0.0 (0.0)	0.0 (0.0)	0.00 (0.00)	0.00 (0.00)	0.00
NI	Nicaragua	Latin America and Caribbean	9.12 (21.08)	9.12 (21.08)	0.00 (0.00)	0.00 (0.00)	0.00
PA	Panama	Latin America and Caribbean	14.19 (13.7)	14.19 (13.7)	0.00 (0.00)	0.00 (0.00)	0.00
PY	Paraguay	Latin America and Caribbean	13.92 (9.31)	13.92 (9.31)	0.00 (0.00)	0.00 (0.00)	0.00
PE	Peru	Latin America and Caribbean	16.46 (16.38)	16.46 (16.38)	0.00 (0.00)	0.00 (0.00)	0.00
PR	Puerto Rico	Latin America and Caribbean	13.93 (16.77)	13.93 (16.77)	0.00 (0.00)	0.00 (0.00)	0.00
BL	Saint Barthelemy	Latin America and Caribbean	6.11 (13.9)	6.11 (13.9)	0.00 (0.00)	0.00 (0.00)	0.00
KN	Saint Kitts and Nevis	Latin America and Caribbean	5.51 (14.29)	5.51 (14.29)	0.00 (0.00)	0.00 (0.00)	0.00
LC	Saint Lucia	Latin America and Caribbean	7.38 (10.85)	7.38 (10.85)	0.00 (0.00)	0.00 (0.00)	0.00
MF	Saint Martin (French Part)	Latin America and Caribbean	4.23 (11.43)	4.23 (11.43)	0.00 (0.00)	0.00 (0.00)	0.00
VC	Saint Vincent and the Grenadines	Latin America and Caribbean	7.77 (12.01)	7.77 (12.01)	0.00 (0.00)	0.00 (0.00)	0.00
SX	Sint Maarten (Dutch part)	Latin America and Caribbean	9.14 (12.89)	9.14 (12.89)	0.00 (0.00)	0.00 (0.00)	0.00
SR	Suriname	Latin America and Caribbean	8.42 (11.03)	8.42 (11.03)	0.00 (0.00)	0.00 (0.00)	0.00
TT	Trinidad and Tobago	Latin America and Caribbean	12.77 (20.43)	12.77 (20.43)	0.00 (0.00)	0.00 (0.00)	0.00
TC	Turks and Caicos Islands	Latin America and Caribbean	9.64 (15.58)	9.64 (15.58)	0.00 (0.00)	0.00 (0.00)	0.00
VI	United States Virgin Islands	Latin America and Caribbean	9.3 (13.22)	9.3 (13.22)	0.00 (0.00)	0.00 (0.00)	0.00
UY	Uruguay	Latin America and Caribbean	9.52 (9.95)	9.52 (9.95)	0.00 (0.00)	0.00 (0.00)	0.00
VE	Venezuela (Bolivarian Republic of)	Latin America and Caribbean	18.13 (17.96)	18.13 (17.96)	0.00 (0.00)	0.00 (0.00)	0.00
FJ	Fiji	Melanesia	7.98 (9.18)	7.98 (9.18)	0.00 (0.00)	0.00 (0.00)	0.00
NC	New Caledonia	Melanesia	5.57 (8.64)	5.57 (8.64)	0.00 (0.00)	0.00 (0.00)	0.00
PG	Papua New Guinea	Melanesia	5.68 (12.13)	5.68 (12.13)	0.00 (0.00)	0.00 (0.00)	0.00
SB	Solomon Islands	Melanesia	4.26 (12.69)	4.26 (12.69)	0.00 (0.00)	0.00 (0.00)	0.00
VU	Vanuatu	Melanesia	3.16 (10.07)	3.16 (10.07)	0.00 (0.00)	0.00 (0.00)	0.00
GU	Guam	Micronesia	10.45 (10.18)	10.45 (10.18)	0.00 (0.00)	0.00 (0.00)	0.00
KI	Kiribati	Micronesia	0.0 (0.0)	0.0 (0.0)	0.00 (0.00)	0.00 (0.00)	0.00
MH	Marshall Islands	Micronesia	0.0 (0.0)	0.0 (0.0)	0.00 (0.00)	0.00 (0.00)	0.00
FM	Micronesia (Federated States of)	Micronesia	0.0 (14.0)	0.0 (14.0)	0.00 (0.00)	0.00 (0.00)	0.00
NR	Nauru	Micronesia	0.0 (0.0)	0.0 (0.0)	0.00 (0.00)	0.00 (0.00)	0.00
MP	Northern Mariana Islands	Micronesia	7.1 (14.57)	7.1 (14.57)	0.00 (0.00)	0.00 (0.00)	0.00
PW	Palau	Micronesia	0.0 (13.86)	0.0 (13.86)	0.00 (0.00)	0.00 (0.00)	0.00
BM	Bermuda	Northern America	8.83 (11.06)	8.83 (11.06)	0.00 (0.00)	0.00 (0.00)	0.00
CA	Canada	Northern America	19.77 (12.15)	19.77 (12.15)	0.00 (0.00)	0.00 (0.00)	0.00
GL	Greenland	Northern America	0.0 (11.04)	0.0 (11.04)	0.00 (0.00)	0.00 (0.00)	0.00
PM	Saint Pierre and Miquelon	Northern America	0.0 (19.23)	0.0 (19.23)	0.00 (0.00)	0.00 (0.00)	0.00
US	United States of America	Northern America	13.99 (16.13)	13.99 (16.13)	0.00 (0.00)	0.00 (0.00)	0.00
AX	Ãland Islands	Northern Europe	6.01 (16.9)	6.01 (16.9)	0.00 (0.00)	0.00 (0.00)	0.00
DK	Denmark	Northern Europe	9.64 (7.77)	9.64 (7.77)	0.00 (0.00)	0.00 (0.00)	0.00
FO	Faroe Islands	Northern Europe	3.52 (8.33)	3.52 (8.33)	0.00 (0.00)	0.00 (0.00)	0.00
GG	Guernsey	Northern Europe	9.61 (13.7)	9.61 (13.7)	0.00 (0.00)	0.00 (0.00)	0.00
IE	Ireland	Northern Europe	17.08 (11.05)	17.08 (11.05)	0.00 (0.00)	0.00 (0.00)	0.00
IM	Isle of Man	Northern Europe	7.66 (12.56)	7.66 (12.56)	0.00 (0.00)	0.00 (0.00)	0.00
JE	Jersey	Northern Europe	13.41 (11.59)	13.41 (11.59)	0.00 (0.00)	0.00 (0.00)	0.00
SJ	Svalbard and Jan Mayen Islands	Northern Europe	0.0 (0.0)	0.0 (0.0)	0.00 (0.00)	0.00 (0.00)	0.00
SE	Sweden	Northern Europe	13.29 (9.84)	13.29 (9.84)	0.00 (0.00)	0.00 (0.00)	0.00
GB	United Kingdom of Great Britain and Northern Ireland	Northern Europe	14.97 (13.02)	14.97 (13.02)	0.00 (0.00)	0.00 (0.00)	0.00
AS	American Samoa	Polynesia	9.0 (23.25)	9.0 (23.25)	0.00 (0.00)	0.00 (0.00)	0.00
CK	Cook Islands	Polynesia	0.0 (12.62)	0.0 (12.62)	0.00 (0.00)	0.00 (0.00)	0.00
PF	French Polynesia	Polynesia	9.96 (13.9)	9.96 (13.9)	0.00 (0.00)	0.00 (0.00)	0.00
WS	Samoa	Polynesia	2.5 (13.39)	2.5 (13.39)	0.00 (0.00)	0.00 (0.00)	0.00
TO	Tonga	Polynesia	0.0 (14.73)	0.0 (14.73)	0.00 (0.00)	0.00 (0.00)	0.00
BN	Brunei Darussalam	Southeastern Asia	12.1 (14.84)	12.1 (14.84)	0.00 (0.00)	0.00 (0.00)	0.00
KH	Cambodia	Southeastern Asia	7.34 (13.49)	7.34 (13.49)	0.00 (0.00)	0.00 (0.00)	0.00
LA	Lao People's Democratic Republic	Southeastern Asia	9.53 (11.99)	9.53 (11.99)	0.00 (0.00)	0.00 (0.00)	0.00
PH	Philippines	Southeastern Asia	16.53 (13.66)	16.53 (13.66)	0.00 (0.00)	0.00 (0.00)	0.00
SG	Singapore	Southeastern Asia	18.48 (16.03)	18.48 (16.03)	0.00 (0.00)	0.00 (0.00)	0.00
TL	Timor-Leste	Southeastern Asia	6.43 (11.93)	6.43 (11.93)	0.00 (0.00)	0.00 (0.00)	0.00
BT	Bhutan	Southern Asia	20.35 (24.65)	20.35 (24.65)	0.00 (0.00)	0.00 (0.00)	0.00
IN	India	Southern Asia	13.65 (21.46)	13.65 (21.46)	0.00 (0.00)	0.00 (0.00)	0.00
MV	Maldives	Southern Asia	11.57 (27.27)	11.57 (27.27)	0.00 (0.00)	0.00 (0.00)	0.00
NP	Nepal	Southern Asia	11.71 (27.27)	11.7 (27.19)	0.0 (0.04)	0.0 (0.0)	0.02
PK	Pakistan	Southern Asia	8.09 (16.06)	8.09 (16.06)	0.00 (0.00)	0.00 (0.00)	0.00
LK	Sri Lanka	Southern Asia	7.4 (15.34)	7.4 (15.34)	0.00 (0.00)	0.00 (0.00)	0.00
GI	Gibraltar	Southern Europe	10.49 (11.63)	10.49 (11.63)	0.00 (0.00)	0.00 (0.00)	0.00
GR	Greece	Southern Europe	20.78 (14.56)	20.78 (14.56)	0.00 (0.00)	0.00 (0.00)	0.00
IT	Italy	Southern Europe	12.73 (12.32)	12.73 (12.32)	0.00 (0.00)	0.00 (0.00)	0.00
SM	San Marino	Southern Europe	5.29 (12.86)	5.29 (12.86)	0.00 (0.00)	0.00 (0.00)	0.00
ES	Spain	Southern Europe	13.3 (9.69)	13.3 (9.69)	0.00 (0.00)	0.00 (0.00)	0.00
BJ	Benin	Sub-Saharan Africa	5.33 (13.28)	5.33 (13.28)	0.00 (0.00)	0.00 (0.00)	0.00
BW	Botswana	Sub-Saharan Africa	15.21 (28.91)	15.21 (28.91)	0.00 (0.00)	0.00 (0.00)	0.00
BF	Burkina Faso	Sub-Saharan Africa	2.88 (6.47)	2.88 (6.47)	0.00 (0.00)	0.00 (0.00)	0.00
BI	Burundi	Sub-Saharan Africa	4.89 (14.36)	4.89 (14.36)	0.00 (0.00)	0.00 (0.00)	0.00
CI	Cote D’Ivoire	Sub-Saharan Africa	5.63 (11.21)	5.63 (11.21)	0.00 (0.00)	0.00 (0.00)	0.00
CM	Cameroon	Sub-Saharan Africa	6.21 (16.74)	6.21 (16.74)	0.00 (0.00)	0.00 (0.00)	0.00
CF	Central African Republic	Sub-Saharan Africa	5.01 (14.9)	5.01 (14.9)	0.00 (0.00)	0.00 (0.00)	0.00
KM	Comoros	Sub-Saharan Africa	3.21 (12.36)	3.21 (12.36)	0.00 (0.00)	0.00 (0.00)	0.00
CG	Congo	Sub-Saharan Africa	4.31 (9.67)	4.31 (9.67)	0.00 (0.00)	0.00 (0.00)	0.00
CD	Democratic Republic of the Congo	Sub-Saharan Africa	6.26 (11.71)	6.26 (11.71)	0.00 (0.00)	0.00 (0.00)	0.00
GQ	Equatorial Guinea	Sub-Saharan Africa	6.52 (18.34)	6.52 (18.34)	0.00 (0.00)	0.00 (0.00)	0.00
ER	Eritrea	Sub-Saharan Africa	0.0 (0.0)	0.0 (0.0)	0.00 (0.00)	0.00 (0.00)	0.00
SZ	Eswatini	Sub-Saharan Africa	13.26 (24.46)	13.26 (24.46)	0.00 (0.00)	0.00 (0.00)	0.00
ET	Ethiopia	Sub-Saharan Africa	6.99 (29.51)	6.99 (29.51)	0.00 (0.00)	0.00 (0.00)	0.00
GA	Gabon	Sub-Saharan Africa	7.58 (22.12)	7.58 (22.12)	0.00 (0.00)	0.00 (0.00)	0.00
GM	Gambia	Sub-Saharan Africa	4.87 (15.49)	4.87 (15.49)	0.00 (0.00)	0.00 (0.00)	0.00
GH	Ghana	Sub-Saharan Africa	5.17 (12.74)	5.17 (12.74)	0.00 (0.00)	0.00 (0.00)	0.00
GN	Guinea	Sub-Saharan Africa	3.86 (11.71)	3.86 (11.71)	0.00 (0.00)	0.00 (0.00)	0.00
KE	Kenya	Sub-Saharan Africa	9.98 (17.57)	9.98 (17.55)	0.0 (0.0)	0.0 (0.0)	3.33
LS	Lesotho	Sub-Saharan Africa	10.7 (19.68)	10.7 (19.68)	0.00 (0.00)	0.00 (0.00)	0.00
LR	Liberia	Sub-Saharan Africa	3.43 (12.58)	3.43 (12.58)	0.00 (0.00)	0.00 (0.00)	0.00
MG	Madagascar	Sub-Saharan Africa	8.97 (11.65)	8.97 (11.65)	0.00 (0.00)	0.00 (0.00)	0.00
MW	Malawi	Sub-Saharan Africa	9.19 (16.99)	9.19 (16.99)	0.00 (0.00)	0.00 (0.00)	0.00
ML	Mali	Sub-Saharan Africa	3.28 (6.96)	3.28 (6.96)	0.00 (0.00)	0.00 (0.00)	0.00
MU	Mauritius	Sub-Saharan Africa	7.43 (12.58)	7.43 (12.58)	0.00 (0.00)	0.00 (0.00)	0.00
YT	Mayotte	Sub-Saharan Africa	3.89 (17.22)	3.89 (17.22)	0.00 (0.00)	0.00 (0.00)	0.00
NA	Namibia	Sub-Saharan Africa	22.0 (28.14)	22.0 (28.14)	0.00 (0.00)	0.00 (0.00)	0.00
NE	Niger	Sub-Saharan Africa	4.03 (10.76)	4.03 (10.76)	0.00 (0.00)	0.00 (0.00)	0.00
NG	Nigeria	Sub-Saharan Africa	4.56 (9.42)	4.56 (9.42)	0.00 (0.00)	0.00 (0.00)	0.00
RE	Reunion	Sub-Saharan Africa	6.72 (11.07)	6.72 (11.07)	0.00 (0.00)	0.00 (0.00)	0.00
RW	Rwanda	Sub-Saharan Africa	10.2 (14.37)	10.2 (14.37)	0.00 (0.00)	0.00 (0.00)	0.00
SH	Saint Helena	Sub-Saharan Africa	10.45 (10.14)	10.45 (10.14)	0.00 (0.00)	0.00 (0.00)	0.00
SN	Senegal	Sub-Saharan Africa	9.59 (14.81)	9.59 (14.81)	0.00 (0.00)	0.00 (0.00)	0.00
SC	Seychelles	Sub-Saharan Africa	6.44 (17.89)	6.44 (17.89)	0.00 (0.00)	0.00 (0.00)	0.00
SL	Sierra Leone	Sub-Saharan Africa	2.2 (9.9)	2.2 (9.9)	0.00 (0.00)	0.00 (0.00)	0.00
SO	Somalia	Sub-Saharan Africa	3.05 (11.02)	3.05 (11.02)	0.00 (0.00)	0.00 (0.00)	0.00
ZA	South Africa	Sub-Saharan Africa	12.8 (17.45)	12.8 (17.45)	0.00 (0.00)	0.00 (0.00)	0.00
TG	Togo	Sub-Saharan Africa	9.09 (18.58)	9.09 (18.58)	0.00 (0.00)	0.00 (0.00)	0.00
UG	Uganda	Sub-Saharan Africa	9.31 (20.55)	9.31 (20.55)	0.00 (0.00)	0.00 (0.00)	0.00
ZM	Zambia	Sub-Saharan Africa	7.37 (14.59)	7.37 (14.59)	0.00 (0.00)	0.00 (0.00)	0.00
ZW	Zimbabwe	Sub-Saharan Africa	8.6 (19.1)	8.6 (19.1)	0.00 (0.00)	0.00 (0.00)	0.00
BH	Bahrain	Western Asia	15.81 (25.38)	15.81 (25.38)	0.00 (0.00)	0.00 (0.00)	0.00
KW	Kuwait	Western Asia	13.25 (20.29)	13.25 (20.29)	0.00 (0.00)	0.00 (0.00)	0.00
AT	Austria	Western Europe	9.19 (9.31)	9.19 (9.31)	0.00 (0.00)	0.00 (0.00)	0.00
BE	Belgium	Western Europe	14.26 (14.35)	14.26 (14.35)	0.00 (0.00)	0.00 (0.00)	0.00
FR	France	Western Europe	10.86 (11.19)	10.86 (11.19)	0.00 (0.00)	0.00 (0.00)	0.00
DE	Germany	Western Europe	9.66 (17.98)	9.66 (17.98)	0.00 (0.00)	0.00 (0.00)	0.00
LI	Liechtenstein	Western Europe	4.91 (9.64)	4.91 (9.64)	0.00 (0.00)	0.00 (0.00)	0.00
LU	Luxembourg	Western Europe	13.47 (11.2)	13.47 (11.2)	0.00 (0.00)	0.00 (0.00)	0.00
NL	Netherlands	Western Europe	8.21 (8.02)	8.21 (8.02)	0.00 (0.00)	0.00 (0.00)	0.00
CH	Switzerland	Western Europe	13.65 (13.14)	13.65 (13.14)	0.00 (0.00)	0.00 (0.00)	0.00
